# Bioconversion of chitin and concomitant production of chitinase and N-acetylglucosamine by novel *Achromobacter xylosoxidans* isolated from shrimp waste disposal area

**DOI:** 10.1038/s41598-020-68772-y

**Published:** 2020-07-17

**Authors:** Kumaran Subramanian, Balamurugan Sadaiappan, Wilson Aruni, Alagappan Kumarappan, Rajasekar Thirunavukarasu, Guru Prasad Srinivasan, Selvaraj Bharathi, Prasannabalaji Nainangu, Pugazhvendan Sampath Renuga, Anandajothi Elamaran, Deivasigamani Balaraman, Mahendran Subramanian

**Affiliations:** 1 School of Bio and Chemical Engineering, Sathyabama Institute of Science and Technology, Chennai, Tamil Nadu 600119 India; 2 Plankton Ecology Laboratory, CSIR- National Institute of Oceanography, Panaji, Goa 403004 India; 3 Sharjah Institute for Medical Research, University of Sharjah, Sharjah, UAE; 4 Centre for Drug Discovery and Development, Sathyabama Institute of Science and Technology, Chennai, Tamil Nadu 600119 India; 5 Centre for Advanced Studies in Marine Biology, Annamalai University, Chidambaram, Tamil Nadu 608502 India; 6 Department of Microbiology, Sri Sankara Arts and Science College, Enathur, Tamil Nadu 631561 India; 7 Department of Zoology - DDE, Annamalai University, Chidambaram, Tamil Nadu 608002 India; 8 Central Aquaculture Genetics Laboratory, Rajiv Gandhi Centre for Aquaculture, Karaimedu, Tamil Nadu 609109 India; 9 School of Medicine, Loma Linda University, Loma Linda, CA 92350 USA; 10 Department of Bioengineering, Department of Computing, Imperial College London, London, SW72AZ, UK

**Keywords:** Applied microbiology, Environmental biotechnology

## Abstract

Marine pollution is a significant issue in recent decades, with the increase in industries and their waste harming the environment and ecosystems. Notably, the rise in shellfish industries contributes to tons of shellfish waste composed of up to 58% chitin. Chitin, the second most ample polymer next to cellulose, is insoluble and resistant to degradation. It requires chemical-based treatment or enzymatic hydrolysis to cleave the chitin polymers. The chemical-based treatment can lead to environmental pollution, so to solve this problem, enzymatic hydrolysis is the best option. Moreover, the resulting biopolymer by-products can be used to boost the fish immune system and also as drug delivery agents. Many marine microbial strains have chitinase producing ability. Nevertheless, we still lack an economical and highly stable chitinase enzyme for use in the industrial sector. So we isolate a novel marine bacterial strain *Achromobacter xylosoxidans* from the shrimp waste disposal site using chitin minimal medium. Placket–Burman and central composite design statistical models for culture condition optimisation predicted a 464.2 U/ml of chitinase production. The culture conditions were optimised for maximum chitinase production recording up to 467 U/ml. This chitinase from the *A. xylosoxidans* was 100% active at an optimum temperature of 45 °C (withstand up to 55 °C) and pH 8 with 80% stability. The HPLC analysis of chitinase degraded shellfish waste reveals a major amino acid profile composition—arginine, lysine, aspartic acid, alanine, threonine and low levels of isoleucine and methionine. These chitinase degraded products and by-products can be used as supplements in the aquaculture industry.

## Introduction

Chitin is made up of N-acetylglucos-amine through a β-(1 → 4) glycosidic bond to assemble poly(β-(1 → 4)-N-acetyl-d-glucosamine (GlcNaC) and it is by far the second most ample biopolymer present in nature. Which is insoluble and resistant to degradation, and it requires chitinase (EC 3.2.1.14) or closely related enzymes for its breakdown. About 10^12^–10^14^ tons of chitin is produced as waste per year^1^ and the major problem for the aquaculture industries is to dispose of this waste. If it is not adequately disposed of or degraded, this waste may cause potential peril to the natural environment and biodiversity^2^. Out of the shellfish industry waste, about 20–58% contains chitin as dry weight^3^.

The best reaction to this issue is to use these losses as inexhaustible crude material for the production of valuable items that can be used in aquaculture, textile, and medical industries^4^. Chitin is in many cases firmly bound with different mixes, for example, lipids, protein and calcium carbonate^5^. Bioconversion of the squandered chitinous waste to valuable chitooligosaccharides involves procedures such as deproteinisation, demineralisation or hydrolysis. These procedures have previously been performed with solid corrosives and bases that incorporates low yields, high levels of expense and consumption issues^6^. The potential substitute for solving this problem is chitin waste management by chitinase^7^.

Glycosyl-hydrolase proteins (EC 3.2.2.14), otherwise known as chitinases can cleave the 1, 4 bonds of the N-acetylglucosamine units, hydrolyse the chitin to chitooligosaccharides [(GlcNAc)n] and act as a catalyst in chitin debasement^8^. Chitosan oligomers (a group of N-deacetylated chitin with different degrees of deacetylation) and other derivatives from chitin degradation by either compound or enzymatic hydrolysis are used in the several fields of the pharmaceutics, horticulture, biotechnology and waste administration. Chitinase is naturally produced by microbes, fungi, plants, insects and animals^9^. Among them, chitinase produced by microbes has received increasing consideration, and potentially fill two needs: (1) they decrease the ecological perils of waste administration, and (2) can increase the worth of the degraded products^10^. Consequently, marine bacterial chitinases are now considered as one of the potential enzymes for this application.

Many marine bacterial genera were reported to produce chitinase like *Alcaligenes*, *Bacillus*, *Vibrio*, *Acinetobacter* and *Streptomyces rimosus*^11^. However, most naturally occurring marine bacteria cannot produce high quantities of chitinase with maximum activity and stability. It is essential to identify, isolate a potential bacterial strain and optimise the culture conditions to maximise the production in a cost-effective manner. As Baas Becking and Beijerincken^12^ mentioned "everything is everywhere, but, the environment selects" most bacterial genera thriving in the shrimp waste disposal site would have the ability to degrade chitin. So, the shrimp waste disposal site should be a potential source for identifying and isolating a suitable chitinase producing bacteria.

For culture condition optimisation, Response Surface Methodology (RSM) is the most broadly used technique^13^. Plackett–Burman (PB) and central composite design (CCD) are multi-purpose examining tools comprising statistical methods for producing empirical models that assist in understanding enzyme kinetics and for optimising the manufacturing conditions of expensive products such as enzymes. The PB design and central composite design were the widely used statistical experimental design in many biotechnological applications^14^, enzyme manufacturing^15^, biomass production^16^ and ethanol production^17^ because of its simplicity and most common saturated design^18^. The unbiased estimation of main effects with the smallest variance can be done using saturated fractional factorial designs. In addition to this, the model is orthogonal, so the impact of individual variables will not interfere with the other variable interactions^19^. Herein, we isolated bacterial strains that produce chitinase from a shrimp waste disposal site. Next, we optimised the culture components using RSM to increase the production of chitinase. Later, the amino acid profile of the chitinase degraded products was determined and discussed.

## Materials and methods

### Colloidal chitin preparation

About 5 g of chitin was mixed with 30 ml of HCl acid (35.5%) and incubated overnight at 4 °C. The colloidal chitin was precipitated by slowly adding 250 ml of chilled ethanol (50%), with constant stirring at 4 °C and left for overnight. The colloidal chitin was centrifuged at 10,000 g for 20 min, and sterile distilled water was used to wash the pellets until the pH value is neutral.

### Isolation of chitinase producing bacteria

The soil samples were collected from five different places of the shrimp and crab waste disposable area near Port Novo landing centre situated on the south-east coast of India (Parangipettai—Lat. 11° 29′; long. 79^o^ 46′ E). The 1 g of soil sample was serially diluted and plated on chitin minimal agar plates (Colloidal chitin, 1%; K_2_HPO_4,_ 0.3 g; NaCl, 4 g; KH_2_PO_4,_ 0.3 g; MgSO_4_·7H_2_O, 0.5 g; Agar, 1.5% per litre). The plates were incubated at 30 °C for 3 days. The bacterial colony showing the maximum zone of chitin clearance was selected for further studies.

### Culture condition for the production of chitinase

A 50 ml of chitin minimal medium (CMM) containing 1% (w/v) colloidal chitin, 0.7% (w/v); KH_2_PO_4_, 0.3% (w/v); K_2_HPO_4_, 4% (w/v); NaCl, 0.5% (w/v); MgSO_4_.7H_2_O and 0.5% (w/v) peptone and pH 7.2 was dispensed in a 250 ml flask and inoculated with 1 ml of young seed culture. The flask was incubated at 37 °C for 3–5 days. Further to incubation, the supernatant was separated via centrifugation of culture broth at 8000 g for 10 min (Sigma laboratory centrifuge 4K15, Chennai, India), and was used as the crude enzyme.

### Chitinase assay

2.5 ml of 1% colloidal chitin (substrate) was mixed with 2.5 ml of PBS, and then 0.5 ml crude enzyme was added; next, the flask was incubated at 45 °C for 1 h. The reaction mixture was added with dinitrosalicylic (DNS) acid and was placed within a boiling water bath for 10 min to stop the reaction. The supernatant was collected by centrifuge, and the reduced sugar was estimated at 540 nm (UV spectrophotometer). Using the GlcNAc standard curve, under assay condition, the quantity of enzyme that yields 1 µmol of reducing sugar/ minute can be defined as one unit of chitinase.

### Characterisation of isolates

The biochemical and morphological characteristics of chitin degrading isolates were characterised, and the strains were identified using Bergey’s manual of determinative bacteriology^20^. The potential bacterial strain was also characterised by 16S rDNA sequencing.

### Molecular characterisation

Genomic DNA was extracted from the bacterial isolates by the phenol–chloroform method. The isolated DNA was visualised using 0.8% agarose gel and quantified using NanoDrop 1000 (Thermo Fisher Scientific, Wilmington, DE, USA). The 16S rDNA sequence of the bacterial strain was amplified by PCR using universal bacterial primers 27F and 1492R and GeNei™ PCR Master Mix (Genei, Bangalore, India). A thermal cycler (Genei, Bangalore, India) was used for the PCR reaction − 94 °C for 5 min (initial denaturation), next step involves 94 °C for 1 min (denaturation), 53 °C for 30 s (annealing), 72 °C for 90 s (elongation)—35 cycles and 72 °C for 7 min (final extension)^21^.

### Phylogenetic analysis

The Genei PCR purification kit (Genei, Bangalore, India) was used in the purification of the amplified PCR products. The sequence of 16s rDNA amplified product was obtained by an automated sanger sequencer (Bioserve, Hyderabad, India). The sequence was edited using BioEdit ver. 7.9^22^ followed by a BLAST search in the NCBI public database to identify the closest sequences. The Phylogenetic tree was drawn in the Mega 6.0 version using the neighbour-joining method^23^. The partial 16S rDNA sequence of bacterial strain was deposited in the GenBank database, and accession numbers were received upon submission to the NCBI database.

### Extraction and purification of chitinase

The crude enzyme was obtained by centrifuging the production medium at 8000 g for 10 min, followed by precipitation of enzymes at different concentration of [50–80% (w/v)] ammonium sulfate slowly added into the supernatant under constant stirring for 30 min at 4 °C. Next, the protein precipitate was collected by centrifugation at 10,000 g for 30 min at 4 °C and dissolved in Tris–HCl buffer with pH.7.5. Next, the enzymes were dialysed at 4–6 °C against 20 mM Tris–HCl buffer with pH 7.5 and incubated for 24 h, and the buffer was changed at every 8 h interval.

The dialysed enzyme solution was then purified with Sephadex 75 column (20 cm × 1.5 cm) equilibrated with 20 mM Tris–HCl buffer with the pH 8.0. The flow rate of 1 ml/5 min was used to elute the enzyme. The eluted fractions were estimated for the chitinase activity. The fractions showing chitinase activity were mixed together and concentrated by using a fast flow DEAE-Sepharose column (1.6 cm × 20 cm) pre-equilibrated with 20 mM Tris–HCl of pH 8 (10 mmol/l). The bounded chitinase was eluted at a flow rate of 400 µl/min using different gradients of NaCl buffer (0–0.5 mol/l) at 4 °C. The collected chitinase fractions were used as an enzyme source for chitinase assays and protein quantification^24^.

### Determination of optimal temperature and pH for enzyme activity

The reaction mixture was incubated at different temperatures and tested for chitinase activity to find the optimum temperature for enzyme activity. To determine the thermal stability of the enzyme, 50 mM Tris–HCl buffer-pH 9.0 along with 0.2 ml of the purified enzyme was incubated for 1 h at a temperature range of 35–95 °C with 5 °C interval. The enzyme activity was evaluated by withdrawing the aliquots for every 15 min and tested using standard chitinase assay. To determine the optimal pH for enzyme activity, 0.2 ml of the purified enzyme along with 50 mM Phosphate buffer was incubated at different pH ranging between 4 and 9 and the residual activity was measured using chitinase assay.

### Identifying the significant variables for maximum chitinase production

The medium components for maximising the chitinase production by *A. xylosoxidans* were screened by using Plackett–Burman design (PBD)^18^. The medium components chosen for the present study include colloidal chitin, yeast extract, peptone, KH_2_PO_4_, FeSO_4_, ZnSO_4_ and MgSO_4_, with each component being represented at two levels, low (− 1) to high (+ 1). Table 1 shows the experimental design. The culture medium was prepared accordingly and incubated at 37 °C for 3–5 days. After incubation, the culture filtrate was centrifuged at 8000 g for 15 min. The actual (measured) and estimated (PBD-based) enzyme values are given in Table 1. The following Eq. (1) calculated the effect of individual parameters on the chitinase production:

1 $${\text{E}}({\text{X}}_{{\text{i}}} ) = 2({\text{M}}_{{\text{i}}}^{ + } - {\text{M}}_{{\text{i}}}^{ - } ){\text{/N}}$$

where *E* is the effect of the parameter*,* $X_{i}$ and $M_{i}^{ + }$ and $M_{i}^{ - }$ are chitinase activity responses, and the number of trials is N.

**Table 1:** Plackett–Burman based experimental design.

Blocks	Yeast extract	Peptone g/l	Colloidal chitin (g/l)	KH_2_PO_4_ g/l	FeSO_4_ g/l	ZnSO_4_ g/l	MgSO_4_ g/l	Enzyme activity (U/ml)	
								Experimental	Predicted
1	1	0.1	50	0.1	0.05	0.05	0.5	218	208.1667
2	1	1	10	1	0.05	0.05	0.1	163.2	178.9
3	0.1	1	50	0.1	0.1	0.05	0.1	212.8	201.8333
4	1	0.1	50	1	0.05	0.1	0.1	179	156.1
5	1	1	10	1	0.1	0.05	0.5	214	198.3
6	1	1	50	0.1	0.1	0.1	0.1	159.2	170.1667
7	0.1	1	50	1	0.05	0.1	0.5	168.4	197.3667
8	0.1	0.1	50	1	0.1	0.05	0.5	203.4	207.1667
9	0.1	0.1	10	1	0.1	0.1	0.1	139.4	129.5667
10	1	0.1	10	0.1	0.1	0.1	0.5	128.2	149.9667
11	0.1	1	10	0.1	0.05	0.1	0.5	200.8	171.8333
12	0.1	0.1	10	0.1	0.05	0.05	0.1	145.2	162.2333

### Optimisation through RSM

The components showing a positive effect on the chitinase production (Table 1), were further optimised using RSM, i.e., we employed a central composite design (CCD) for maximum production of chitinase enzyme. The significant variables for chitinase production, i.e. peptone, chitin, KH_2_PO_4_ and MgSO_4_ and a total of 31 experiments were performed with these variables at five different levels (− 2, − 1, 0, + 1, and + 2). The full experimental plan, including the values of the significant variables, is given in Table 2. The enzyme activity U/ml (response value) was obtained by calculating the average of the triplicate in each trial. Analysis of variance (ANOVA) was performed on the data obtained from RSM on Chitinase production. The response surface regression involves the polynomial equation (2):

2 $${\text{Y}} = \beta_{0} + \sum_{{\text{i}}} \beta_{ii } X_{i} + \sum_{{{\text{i}} }} \beta_{ii } X^{2}_{i} + \sum_{{{\text{i}} }} \beta_{ij } X_{i} X_{{\text{j}}} \quad{\tilde{\text{u}}}^{\prime } \beta$$

**Table 2:** CCD based experimental design and results.

Run order	Peptone g/l	Chitin g/l	KH_2_PO_4_ g/l	MgSO_4_ g/l	Enzyme activity U/ml	
					Experimental	Predicted
1	0.325	20	0.325	0.2	403	399.5833
2	0.775	20	0.325	0.2	390	385.5417
3	0.325	40	0.325	0.2	377	378.5417
4	0.775	40	0.325	0.2	405	404.25
5	0.325	20	0.775	0.2	383	383.2083
6	0.775	20	0.775	0.2	388	388.9167
7	0.325	40	0.775	0.2	397	394.9167
8	0.775	40	0.775	0.2	437	440.375
9	0.325	20	0.325	0.4	439	434.2083
10	0.775	20	0.325	0.4	386	388.9167
11	0.325	40	0.325	0.4	398	397.9167
12	0.775	40	0.325	0.4	394	392.375
13	0.325	20	0.775	0.4	352	353.5833
14	0.775	20	0.775	0.4	331	328.0417
15	0.325	40	0.775	0.4	347	350.0417
16	0.775	40	0.775	0.4	360	364.25
17	0.1	30	0.55	0.3	372	373.7083
18	1	30	0.55	0.3	375	373.875
19	0.55	10	0.55	0.3	373	377.7083
20	0.55	50	0.55	0.3	397	392.875
21	0.55	30	0.1	0.3	416	421.0417
22	0.55	30	1	0.3	381	376.5417
23	0.55	30	0.55	0.1	407	409.0417
24	0.55	30	0.55	0.5	369	367.5417
25	0.55	30	0.55	0.3	459	462
26	0.55	30	0.55	0.3	463	462
27	0.55	30	0.55	0.3	466	462
28	0.55	30	0.55	0.3	462	462
29	0.55	30	0.55	0.3	459	462
30	0.55	30	0.55	0.3	467	462
31	0.55	30	0.55	0.3	458	462

where *Y* is the prediction, *β*_0_ is the constant term, *β*_*i*_ is the *i*th linear coefficient, *β*_*ii*_ is the *i*th quadratic coefficient, *β*_*ij*_ is the *ij*th interaction coefficient, and *X*_*i*_,* X*_*j*_* are* independent variables. The independent variables were expressed as *X*1, *X*2, *X*3, *X*4, *X*5 and can be represented as the second-order polynomial equation (3):

3 $${\text{Y}} = \beta_{0} + \sum_{{{\text{i}} = 1}}^{{\text{k}}} \beta_{i } X_{i} + \sum_{{{\text{i}} = 1}}^{{\text{k}}} \beta^{ii} X_{i}^{2} + \sum_{{\text{i}}} \sum_{{\text{j}}}^{{\text{k}}} \beta_{j} X_{i} X_{j + \epsilon }$$

Fisher's check was used to evaluate the statistical importance of the equation version and model terms. The quality of fit for the second-order polynomial equation was expressed through the coefficient of determination (R^2^) and the adjusted R^2^. The fitted polynomial equation (3) was expressed as 3-dimensional surface plots to visualise the relationship between the responses and the utilised variables. Point optimisation approach was deployed to optimise each variable for the maximum reaction^25^.

### Amino acid analysis

The colloidal chitin was mixed with 500 µl of purified chitinase and incubated at 45 °C for 1 h. Subsequent to incubation, the centrifugation of the reaction mixture was performed at 3500 g for 15 min. 1 N NaOH was used to neutralise the filtered supernatant. The neutralised supernatant was diluted to 100 times of the volume by adding milli-Q water, and 20 µl was injected into HPLC (Shimadzu, USA) containing LC1oAT HPLC pumps. The analysis was carried out with 30% milli-Q water and 70% acetonitrile at a flow rate of 1000 µl/min in 40 °C as mobile phase and detected by SPD-M10A detector with UV absorbance at 250 nm.

### Ethical approval

No animal or human studies were carried out by the authors.

## Results and discussion

### Isolation and characterisation of chitin producing strains

**Figure 1:**
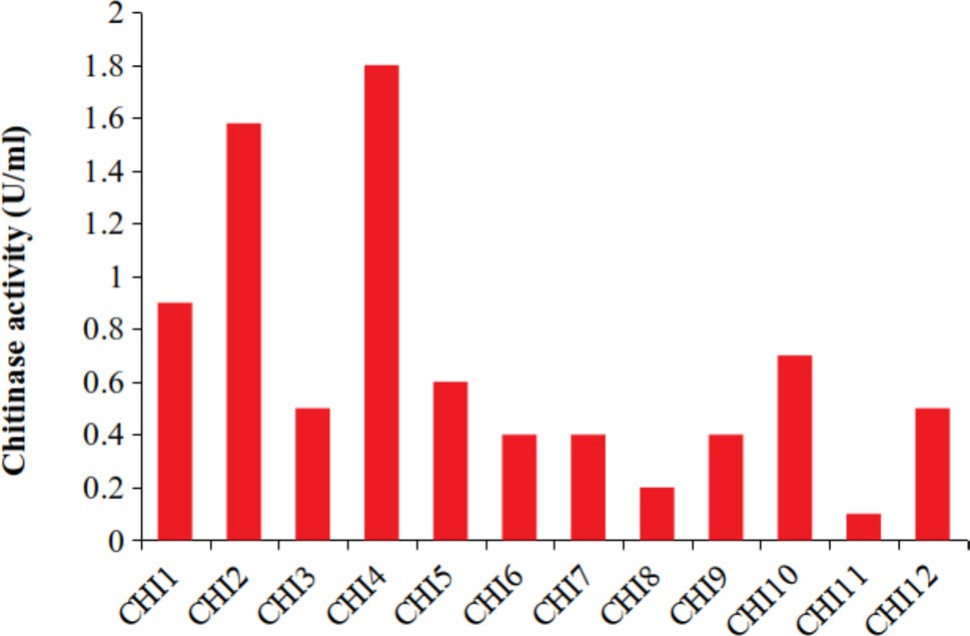
Screening of potential strains of chitinase producing bacteria.

**Figure 2:**
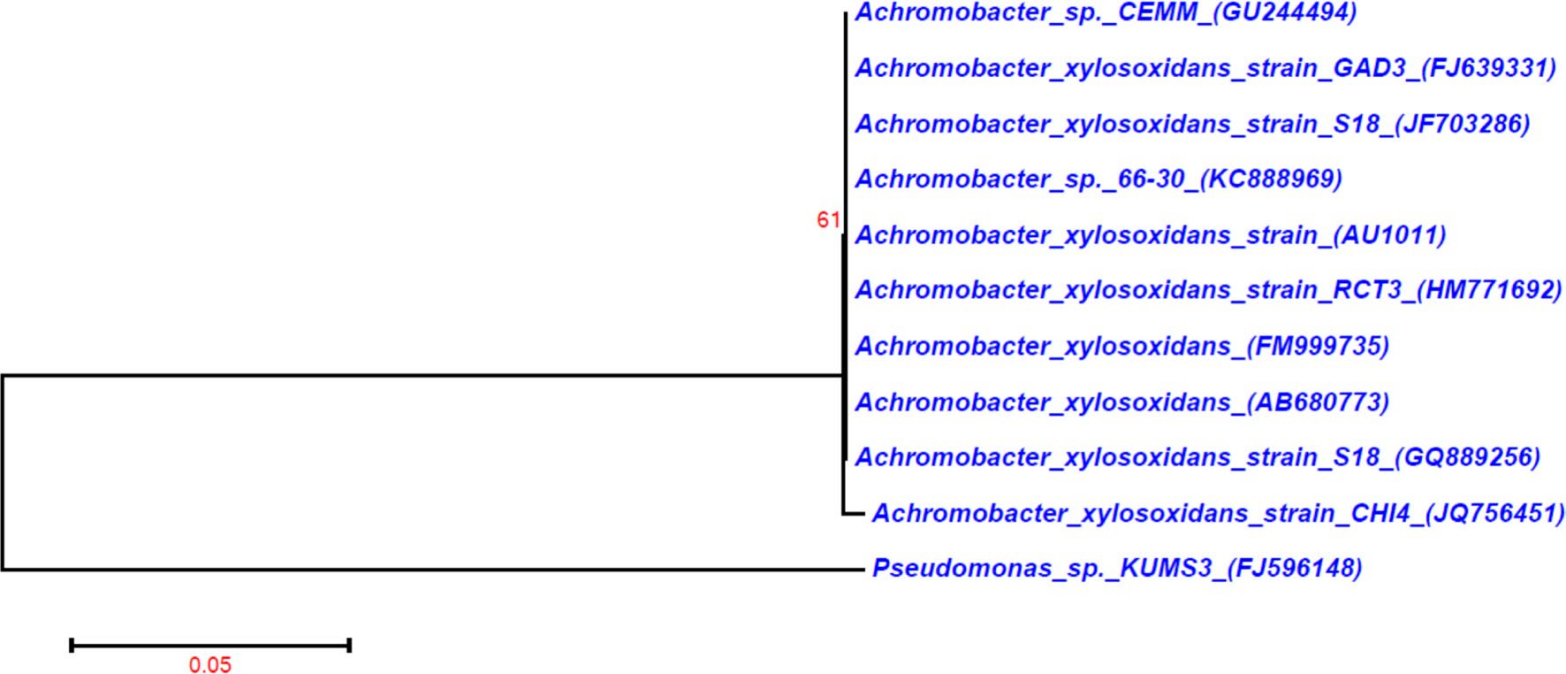
Phylogenetic tree of *A. xylosoxidans* with *Pseudomonas *sp. KUMS3 as outgroup.

In this present investigation, the isolation and characterisation of potent chitin degrading bacteria from the marine environment were performed. Fourteen bacterial strains were isolated from the shrimp waste disposal site, among the 14 strains one bacterial strain showing maximal chitinase activity (1.8 U/ml, Fig. 1) was selected, and this strain was named as chitinase producing bacterial strain 4 (CHI4). The morphological and biochemical test showed this strain CHI4 was rod-shaped, Gram-negative, motile bacteria, utilising catalase and oxidase and oxidise sugars such as xylose and glucose. The 1091 bp 16S rDNA sequence and blast analysis showed that this bacterial strain was closely similar to *Achromobacter xylosoxidans.* Moreover, 99 per cent similar to the *Achromobacter xylosoxidans* strain Osb (Genbank accession no: MN889379.1) and *Achromobacter xylosoxidans* strain Spa05NA (Genbank accession no: MT052658.1). The accession number in the NCBI database for the deposited 16S rDNA partial sequence was JQ756451. The phylogenetic tree for the selected strains was constructed using the neighbour-joining method (Fig. 2). Earlier, this species was named as *Alcaligenes xylosoxidans*. Later, based on the 16S rDNA sequence, it was renamed to *Achromobacter xylosoxidans*^26^. It is widely found in soil, water, dialysis and chlorhexidine solutions, sometimes present in the gastrointestinal and respiratory tract of humans^27^.

In the past, numerous marine bacterial strains were reported to produce chitinase such as *Bacillus cereus* SV1^28^, *Alteromonas* sp. O-7^29^, *Aeromonas hydrophila*^30^, *Pseudoalteromonas *sp. DC14^31^, *Pseudoalteromonas* sp. DXK012^32^, *Pseudomonas aeruginosa* K-187^3^, *Sphingomonas *sp. CJ-5^33^, and *Serratia marcescens*^7^. A closely related study we were able to find was on an *Alcaligenes xylosoxidans* strain isolated from seafood industry waste^34,35^.

### Chitinase purification

The chitinase enzyme was effectively precipitated from the CHI4 culture supernatant system by salting-out method at 75% concentration of ammonium sulfate. After the dialysis and Sephadex-75 column chromatography purification, seven fractions containing enzyme activity were obtained. The enzyme specific activity increased to 10.75 U/mg after threefold purification using the Sephadex column, and the protein recovered was 8 mg (Table 3) and a total activity of 86 U/mg (Yield-12%) of chitinase enzyme were obtained. However, a high amount of recovery rate with 27% yield was noted in *Vibrio* sp.^36^.

**Table 3:** Estimation of *A. xylosoxidans* enzyme activity.

	Protein (mg)	Total activity (U)	Specific activity (U/mg)	Purification fold	Recovery %
Culture supernatant	200	716	3.58	1.0	100
Ammonium sulphate precipitate	65	351	5.4	1.50	49
Sephadex column purified	8	86	10.75	3	12

### Effect of temperature and pH on the stability and activity of chitinase

**Figure 3:**
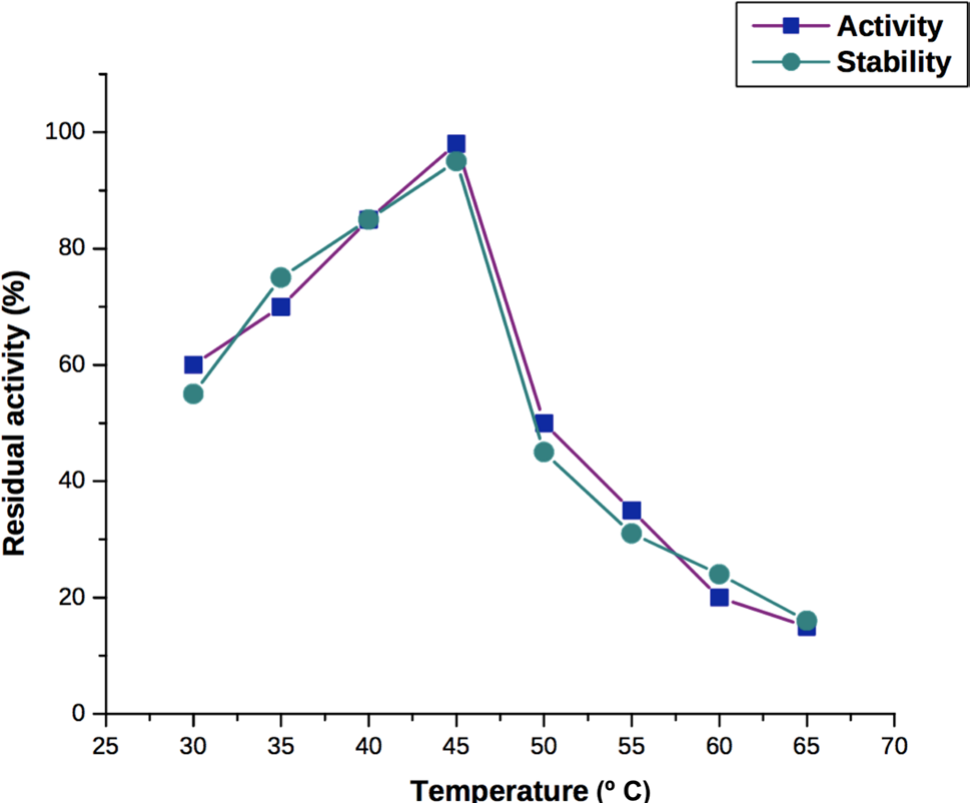
Effect of temperature on activity and stability of purified chitinase from *A. xylosoxidans.*

The purified chitinase enzyme obtained from *A. xylosoxidans* was active 100% at an optimum temperature of 45 °C. The enzyme activity was observed at temperatures above 45 °C, but it decreased to 45% at 50 °C and lost its activity above 65 °C (Fig. 3). The optimum temperature for chitinase activity in various marine bacteria was reported in the past, i.e. 55 °C for *Bacillus cereus* SV1^28^, 50 °C for *Alcaligenes xylosoxydans*^35^ and *Alteromonas* sp. O-7^29^, 40 °C for *Aeromonas hydrophila*^30^, *Pseudoalteromonas *sp. DC14^31^, *Pseudoalteromonas* sp. DXK012^32^, *Pseudomonas aeruginosa* K-187^3^ and 36 °C for *Sphingomonas *sp. CJ-5^33^ , 30 °C for *Serratia marcescens*^7^. However, in the present study, the optimum temperature for chitinase activity ranged between 30–50 °C and was able to withstand up to 65 °C.

**Figure 4:**
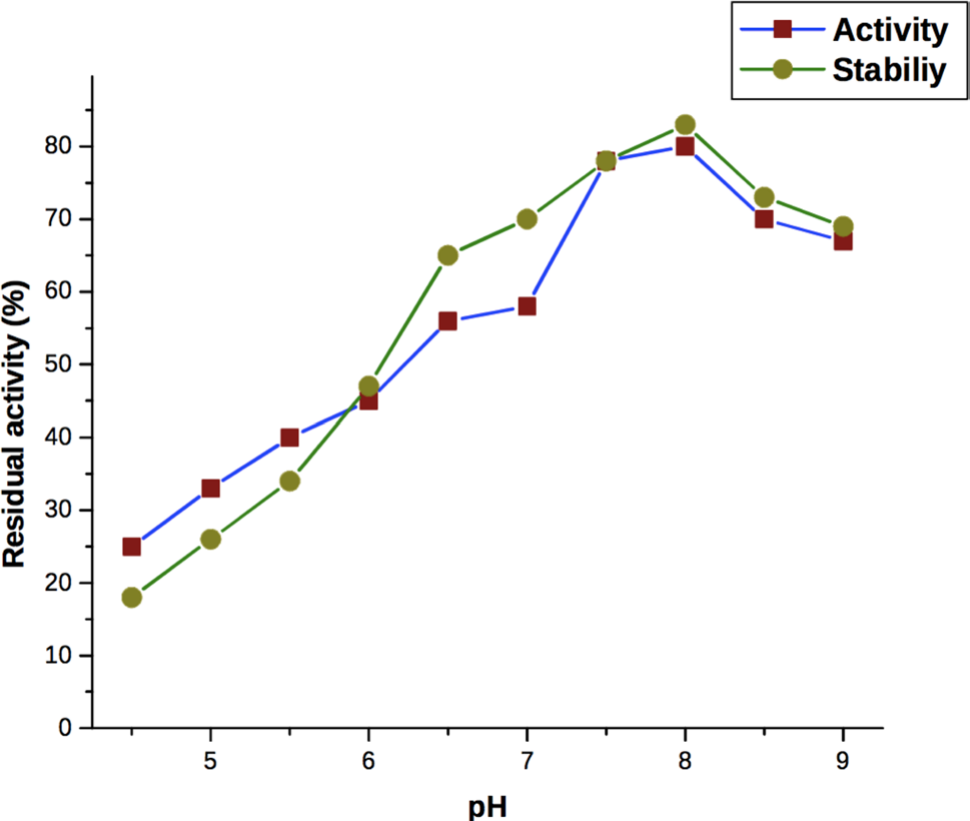
Effect of pH on activity and stability of purified chitinase from *A. xylosoxidans.*

Chitinase from *A. xylosoxidans* was active in pH ranging between 7 and 9 and was found to have an optimum pH of 8.0. Moreover, the enzyme was 83% stable and 80% active at pH 8.0 (Fig. 4). The activity of chitinase reduced at pH beyond and below 6 due to the changes of ionic residues in the protein molecules. The ionic amino acid residue, which is found at the active site of the protein, is critical to maintaining protein conformation structure. Our results compared with the chitinase produced by the *Alteromonas* sp. O-7^29^. Also, the *Streptomyces chilikensis* RC1830 produced chitinase was active between pH 5–8 with an optimum pH 7^37^. The activity of chitinase produced from certain marine bacterias like *Sphingomonas *sp. CJ-5^33^ and *Serratia marcescens*^7^ has an optimum pH of 7, and the optimum pH was 9 for *Pseudoalteromonas *sp. DC14^31^. Whereas, chitinase of *Alcaligenes xylosoxydans* was active in acidic pH^35^ and herein, *A. xylosoxidans* chitinase was active in a wide range of pH from neutral to base with 80% activity.

### Screening of significant variables by Plackett–Burman design

**Figure 5:**
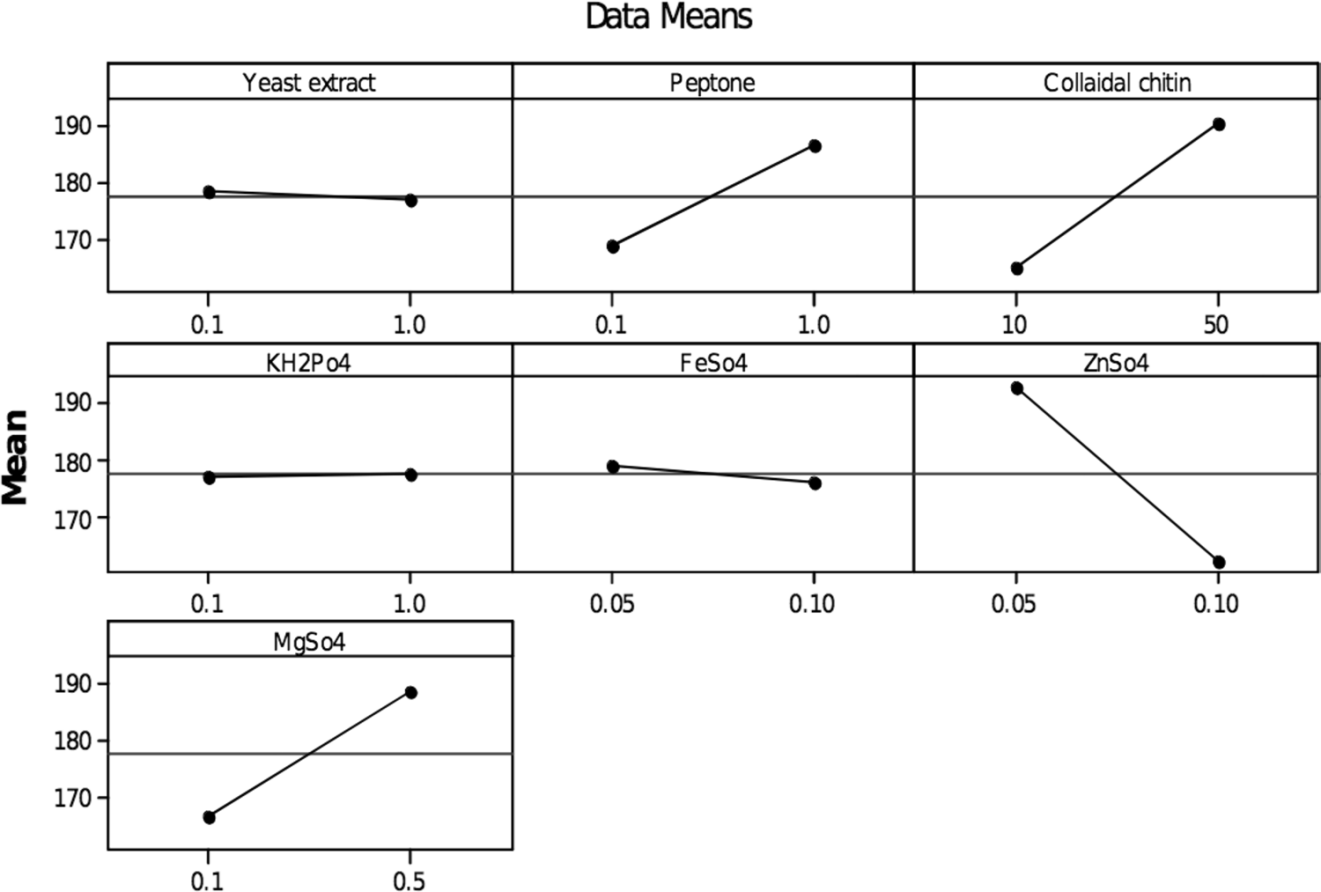
Main effects plot for chitinase production.

Totally twelve variables were selected for analysing the effects on chitinase production by *A. xylosoxidans* using the Plackett–Burman design. The variables showing a positive impact on the enzyme production were selected for the optimisation study, and the significant variables of chitinase production with corresponding responses are exposed in Table 1. Besides, colloidal chitin, with an effect of 25.0, MgSO_4_ (22.33) was the next significant factor to influence chitinase production, followed by peptone (17.53) and KH_2_PO_4_ (0.53), while the other variables, Yeast extract (− 1.40), FeSO_4_ (− 2.93) and ZnSO_4_ (− 16.04) had a negative impact on the chitinase production (Fig. 5 and Table 4). Significant variables were identified, i.e., Colloidal chitin, peptone, KH_2_PO_4_ and MgSO_4_ were selected along with the incubation period (37 °C for 3–5 days) to determine the optimum chitinase production by CCD further.

**Table 4:** Result of the Plackett–Burman design based screening experiment.

Term	Effect	Coefficient
Constant		177.63
Yeast extract	− 1.40	− 0.70
Peptone	17.53	8.77
Colloidal chitin	25.00	12.50
KH_2_Po_4_	0.53	0.27
FeSo_4_	− 2.93	− 1.47
ZnSo_4_	− 30.27	− 15.13
MgSo_4_	22.33	11.17

Similar experimental variables such as yeast extract, urea, KH_2_PO_4_ and colloidal chitin, were found to influence chitinase production by *Paenibacillus* sp.^38^, however, in the present study peptone showed a positive impact. Colloidal chitin, syrup of date, yeast extract, K_2_HPO_4_ and KH_2_PO_4_ showed a positive impact on chitinase production in *Streptomyces griseorubens* C9^39^. The colloidal chitin as the sole carbon source, showed enhanced chitinase production in *Microbispora* sp.^40^. In contrast, better chitinase production was observed in the marine bacteria, *Aeromonas hydrophila* SBK1 when shrimp shell powder was used rather than colloidal chitin^41^. Phosphate was reported to have a negative effect on the chitinase production in *Streptomyces griseus*^42^, but herein, it did have a modest positive effect on chitinase production (K_2_HPO_4_).

Peptone and yeast extract, the nitrogen sources (organic) did increase our chitinase production. Similar to the studies demonstrating chitinase production from *Alcaligenes xylosoxydans*^34^, *Serratia marcescens*^43^ and *Paenibacillus* sp. D1^38^. In contrast to this, *Paenibacillus *sp. AD showed maximum chitinase synthesis when ammonium sulfate was used as a nitrogen source^44^, while sodium nitrate was ideal for the production of chitinase in *Stachybotrys elegans*^45^. In the present study, KH_2_PO_4_ and MgSO_4_ showed a positive impact on chitinase production, whereas, their influence was insignificant during chitinase production by *Pseudomonas fluorescens* strain HN1205^46^.

### Optimisation of significant variables by CCD

The CCD experiments were conducted with four different variables (peptone, chitin, KH_2_PO_4_, MgSO_4_) to determine their optimum concentration for the maximum production of chitinase. The ANOVA analysis for this model is presented in Table 5, and the *P* value for the model was 0.0001, *P* value of lack of fit was 0.260, suggesting that the data was a good fit with the model.

**Table 5:** Analysis of variance for chitinase production.

Source	DF	Seq SS	Adj SS	Adj MS	F	*P*
Regression	14	45,770.8	45,770.8	3269.34	177.47	0.0001
Linear	4	5898.8	5898.8	1474.71	80.05	0.0001
Square	4	31,492.1	31,492.1	7873.02	427.37	0.0001
Interaction	6	8379.9	8379.9	1396.65	75.81	0.0001
Residual error	16	294.7	294.7	18.42		
Lack-of-fit	10	218.8	218.8	21.88	1.73	0.260
Pure error	6	76.0	76.0	12.67		
Total	30	46,065				

**Figure 6:**
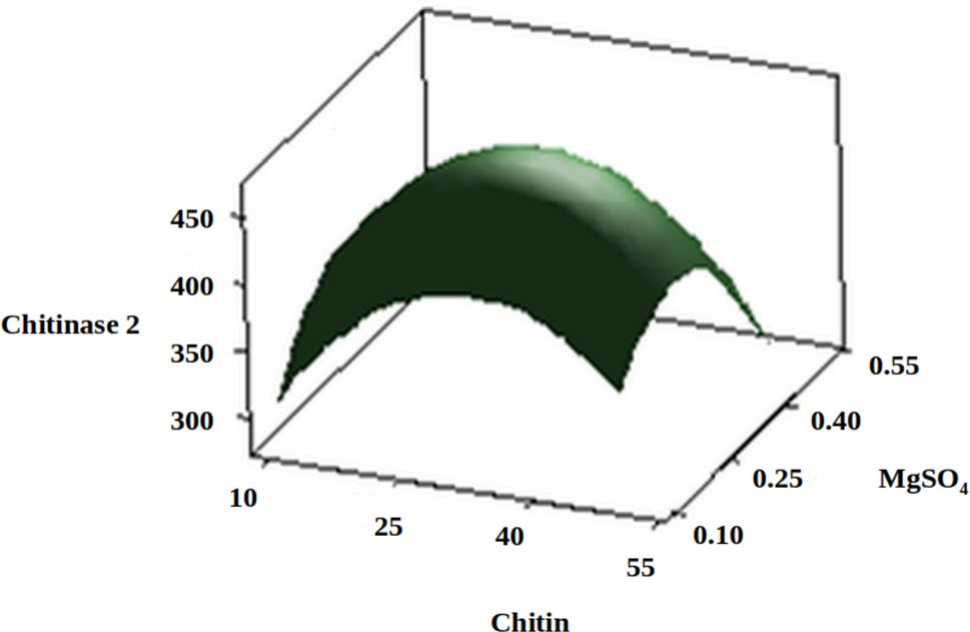
Response surface curve showing the effect of chitin and MgSO_4_ on chitinase production by *A. xylosoxidans*.

**Figure 7:**
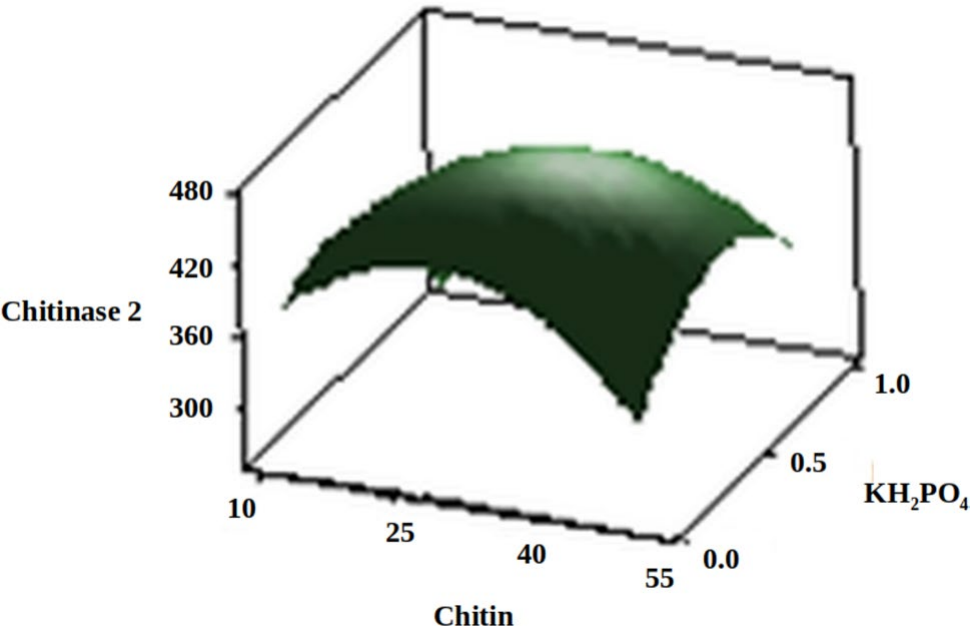
Response surface curve showing the effect of chitin and KH_2_PO_4_ on chitinase production by *A. xylosoxidans*.

**Figure 8:**
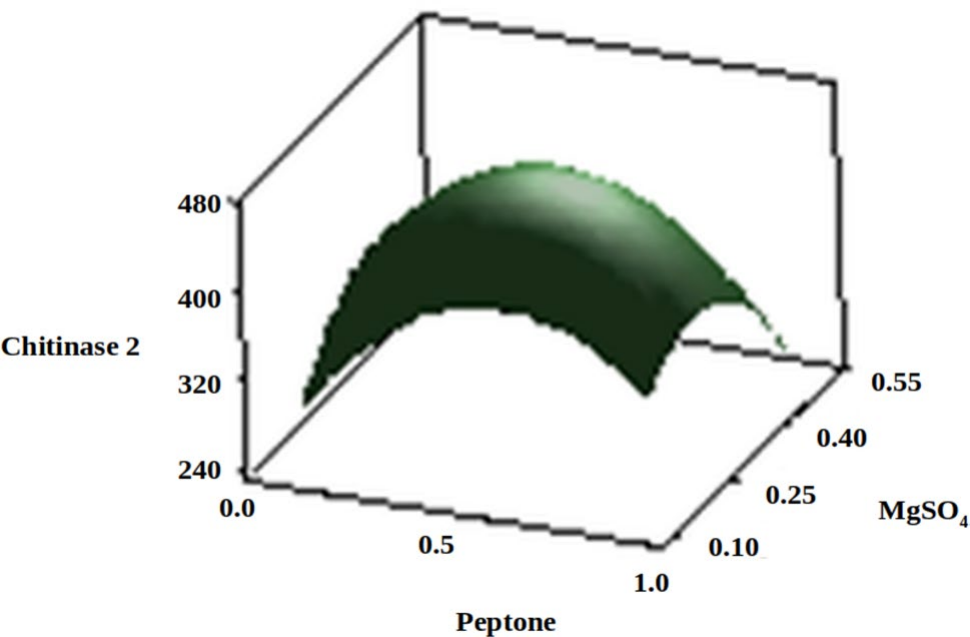
Response surface curve showing the effect of peptone and MgSO_4_ on chitinase production by *A. xylosoxidans*.

**Figure 9:**
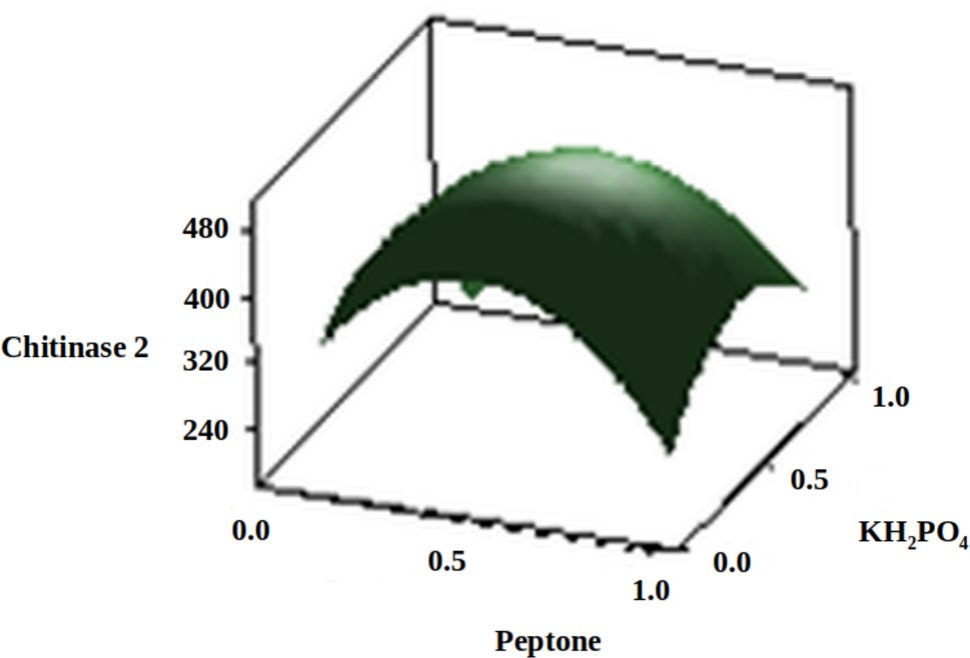
Response surface curve showing the effect of peptone and KH_2_PO_4_ on chitinase production by *A. xylosoxidans*.

**Figure 10:**
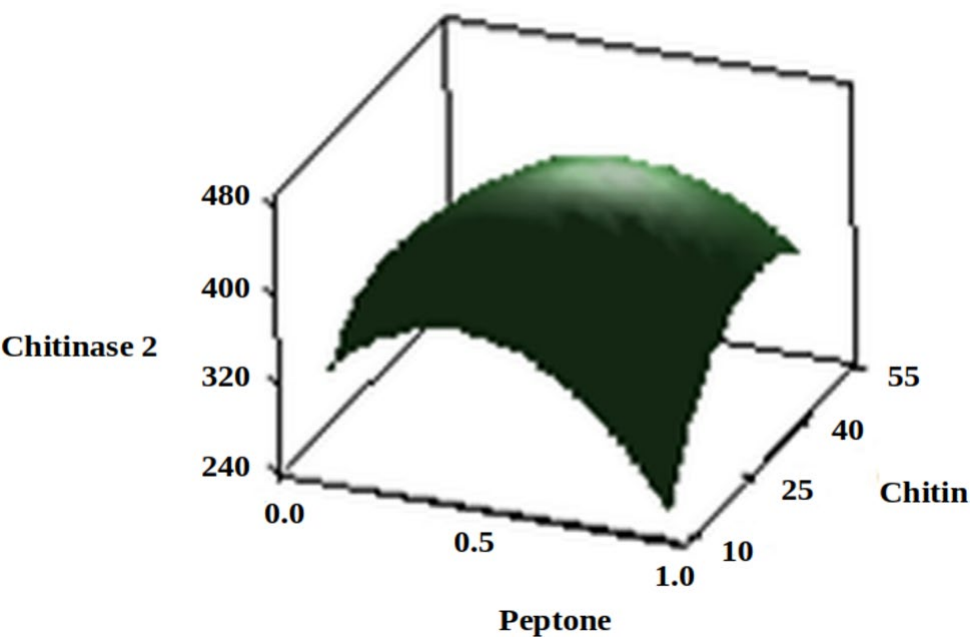
Response surface curve showing the effect of peptone and chitin on chitinase production by *A. xylosoxidans*.

**Figure 11:**
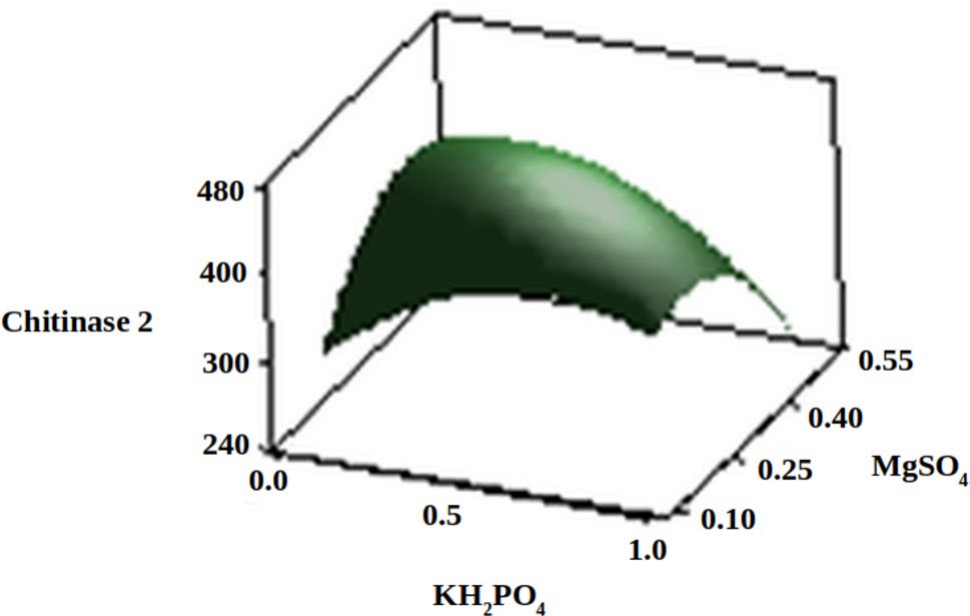
Response surface curve showing the effect of KH_2_PO_4_ and MgSO_4_ on chitinase production by *A. xylosoxidans*.

**Figure 12:**
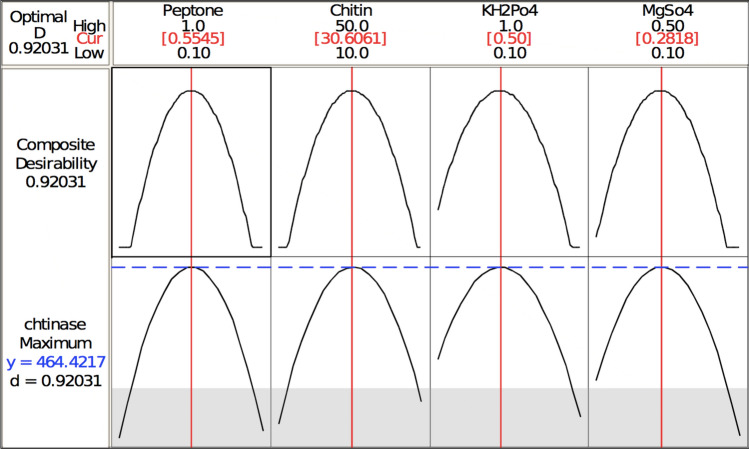
The predicted optimum values of peptone, chitin, KH_2_PO_4_ and MgSO_4_ for chitinase production.

The three-dimensional response surface plots (regression equation in graphical representations) are shown in Figs. 6, 7, 8, 9, 10 and 11. The primary objective of the response surface is to ensure the variables’ optimum values response is maximised powerfully. The surface plots affirm that the objective function was unimodal, which showed an optimum in the centre, and that the model is in a good prediction of the experiment. The main optimum point was estimated by the gradient method with the direction of the medium was the steepest rise for the chitinase production and estimated from the surface plots. The optimal values of peptone, chitin, KH_2_PO_4_ and MgSO_4_ were estimated in actual units, and they were 0.5545 g/l, 30.6061 g/l, 0.50 g/l and 0.2818 g/l, respectively, with a chitinase activity of 464.2 U/ml (predicted). To confirm the predicted values, experiments were performed with these predicted conditions, and the recorded chitinase activity was 467 U/ml (Fig. 12). This activity was slightly high when compared to the predicted value. Such studies reiterate the fact that CCD is a highly reliable model.

Furthermore, the recorded chitinase activity was also higher than the chitinase production observed in most marine bacteria isolated from shrimp shell waste. A review on chitinase production and the produced quantity by isolated marine bacterial strains can be seen in Table 6. Whereas, one should note that chitinase production from the marine fungi *Aspergillus flavus* MK20 isolated from coastal sediment can produce chitinase up to 620.5 U/ml, with RSM (Box-Behnken Design-BBD) based estimation up to 1520 U/ml^47^.

**Table 6:** Review on chitinase production and the recorded quantity by isolated marine bacterial strains.

Species	Source of isolation	Quantity in U/ml	RSM-quantity in U/ml
*Streptomyces* sp.^49^	Shrimp shell waste	–	31.62
*Bacillus cereus* SV1^28^	Fishing port	–	82.8
*Aeromonas hydrophila*^41^	Shrimp shell waste	–	21.4 (BBD)
*Pseudoalteromonas* sp. DC14^31^	Caspian Sea	2.30	21.90 U/dl (BBD)
*Bacillus pumilus*^50^	Mangrove soil	3.36	23.19
*Streptomyces griseorubens C9*^39^	Semi-arid soil	–	9.7 (PBD and CCD)
*Bacillus* sp*.* CH-2^51^	Fish market soil	–	3.1 (PBD and CCD)
*Paenibacillus* sp.^44^	Coastal soil	3.84	20.01 (PBD and CCD)
*Paenibacillus elgii*^52^	Marine soil	3.157	24.53
*Pseudomonas fluorescens* strain HN1205^46^	Coastal soil	–	1.03 U/mL(PBD and CCD)
*Paenibacillus* sp. D1^38^		–	93.2 (PBD and CCD)

Optimisation of *Paenibacillus *sp. D1, a marine microbial produced chitinase was demonstrated using CCD (predicted 85.8 U/ml) with an experimental design including 30 different formulations yielded 93.2 U/ml^38^. Whereas, herein, the maximum similarity was recorded between the experimental and the predicted results. Similar studies have also reported a positive effect of using statistical methods such as CCD to increase chitinase production by 141% in *Alcaligenes xylosoxidans*^35^.

### Amino acid analysis of chitin degraded product

**Figure 13:**
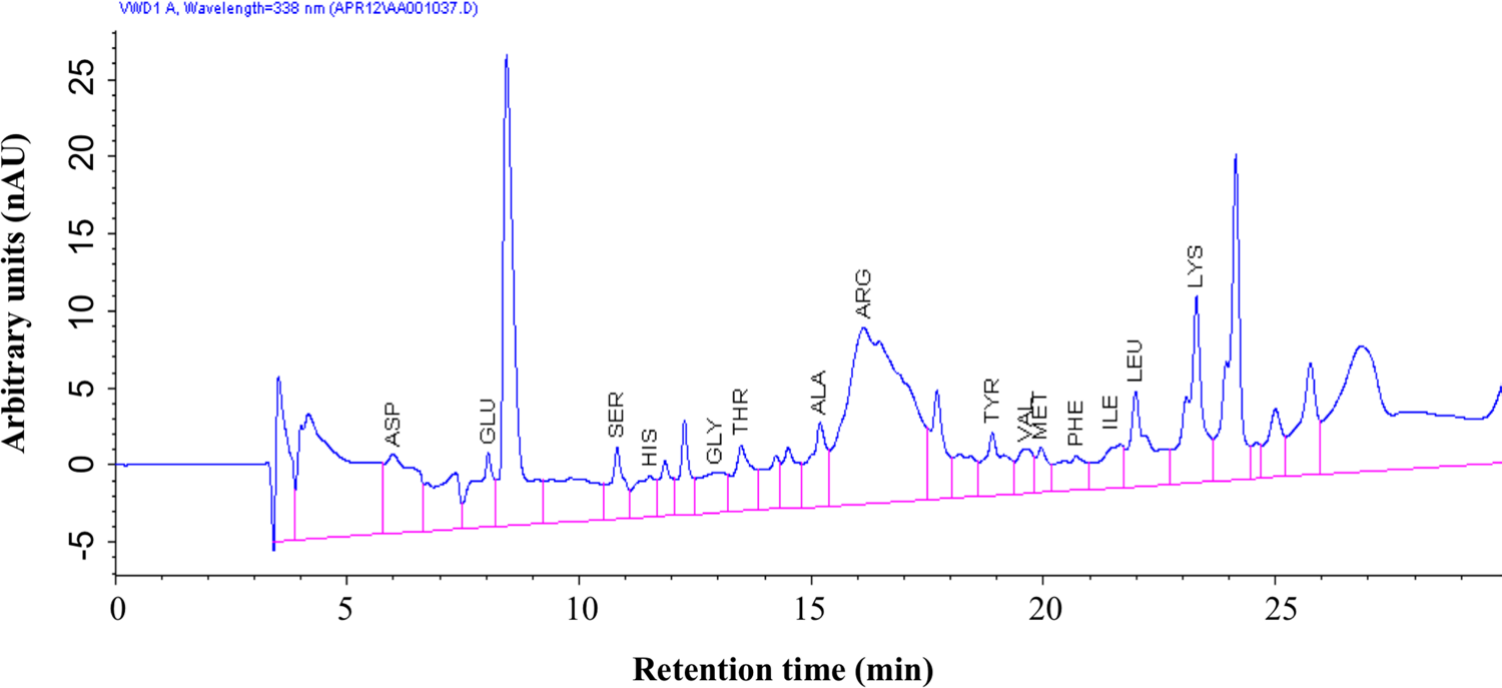
Amino acid composition of chitin degraded product by HPLC analysis.

The chitin degraded end products were analysed by HPLC. Usually, the chitin derivatives are oligomeric and monomeric polysaccharides, and few proteins are present. The results showed that in total, 15 amino acids were present (Fig. 13 and Table 7). The primary amino acid profile composition of the chitin degraded products includes lysine, aspartic acid, arginine, and low levels of isoleucine and methionine. Presence of major amino acids within the derivatives makes it an ideal aquaculture supplement.

**Table 7:** Estimated amino acid composition of chitin degraded product.

Amino acids	Chitinase degraded product (ng/ml)
Aspartic acid	23.9
Glutamic acid	14.7
Serine	12.7
Histidine	9.2
Glycine	11.5
Threonine	15.6
Alanine	17.5
Arginine	61.3
Tyrosine	13.0
Valine	15.2
Methionine	10.9
Phenylalanine	11.0
Isoleucine	9.1
Leucine	19.7
Lysine	37.6

While most prior studies focused on the amino acid profile of the chitinase enzyme, we have investigated the amino acid profile of chitin degraded products. Which lead to an exciting find that the amino acid profile of chitin degraded products has more composition along with traces of chitinase when compared with the amino acid profile of chitinase enzyme. Likewise, asparagine, threonine, glycine and alanine were reported as the major amino acids in chitin derivatives^48^.

## Conclusion

In conclusion, we isolated 14 different strains of marine bacteria capable of degrading chitin. Out of that, the bacterial strain that had high chitinase activity was isolated. We have demonstrated that this *Achromobacter xylosoxidans* strain isolated from a shrimp waste disposal site can produce chitinase, which in turn can degrade chitin along with the concomitant production of N-acetyl-d-glucosamine. The simultaneous production of value-added bioproducts makes this an eco-friendly solution for environmental pollution caused by shrimp waste disposable. Moreover, these end products could also be promoted as a cost-effective supplement for the aquaculture industry. Furthermore, we have also demonstrated that this chitinase production by *Achromobacter xylosoxidans* can be optimised using RSM techniques.
